# Signatures of adaptation in myopia-related genes on the sunlight exposure hypothesis

**DOI:** 10.1186/s40101-023-00341-4

**Published:** 2023-11-02

**Authors:** Tian Xia, Kazuhiro Nakayama

**Affiliations:** Department of Integrated Biosciences, Graduate School of Frontier Sciences, The University of Tokyo, Kashiwa, 277-8562 Japan

**Keywords:** Myopia, Adaptation, Gene-environment interaction

## Abstract

**Background:**

Myopia is a common eye disorder that results from gene-environment interactions. The prevalence of myopia varies across populations, and exposure to bright sunlight may prevent its development. We hypothesize that local adaptation to light environments during human migration played a role in shaping the genetic basis of myopia, and we aim to investigate how the environment influences the genetic basis of myopia.

**Method:**

We utilized the whole-genome variant data of the 1000 Genomes Project for analysis. We searched myopia-associated loci that were under selection in Europeans using population branch statistics and the number of segregating sites by length statistics. The outliers of these statistics were enriched in the Kyoto Encyclopedia of Genes and Genomes pathways and the gene ontology biological process terms in searching for pathways that were under selection. We applied Bayesian inference to estimate the correlation between environmental factors and allele frequencies of the selected loci and performed causal inference of myopia using two-sample Mendelian randomization analysis.

**Results:**

We detected signatures of adaptation in vision and light perception pathways, supporting our hypothesis of sunlight adaptation. We discovered a strong correlation between latitude and allele frequencies in genes that are under significant selection, and we found pleiotropic effects of pigmentation or circadian rhythm genes on myopia, indicating that sunlight exposure influences the genetic diversity of myopia.

**Conclusions:**

Myopia genes involved in light perception showed signs of selection. Local adaptation during human migration shaped the genetic basis of myopia and may have influenced its global prevalence distribution.

**Supplementary Information:**

The online version contains supplementary material available at 10.1186/s40101-023-00341-4.

## Introduction

Myopia, or nearsightedness, is widely acknowledged as the leading cause of distance vision impairment worldwide [1]; since its emergence in the last century, it has become a significant public health burden [2, 3] and is believed to result from gene-environment interactions [4, 5]. Previous studies have demonstrated that spending more time outdoors and under bright sunlight can help prevent the progression [6, 7]. This protective effect in both human and animal models [8–10] suggests that the gene-environment interactions of sunlight in myopia are evolutionarily conserved.

Findings in previous genetic studies of myopia [4, 11] suggested that genetic susceptibility to myopia was not different between Europeans and East Asians. In contrast, the prevalence of myopia was significantly higher in East Asia than in Europe (Supplementary Figure S1) [1, 12–15] despite adequate urbanization and education levels. Myopia is considered to be an evolutionary mismatch [16], which was hypothesized to be the result of genes that were advantageous in the ancient environment and became deleterious in the modern environment [17]. Bright sunlight inhibits myopia progression and profoundly influences genetic and phenotypic diversity in humans [18, 19]. We hypothesized that adaptations to different sunlight environments during modern human migration diversified the genetic basis of myopia worldwide and subsequently contributed to discrepancies in the prevalence of myopia. Major large-scale genetic studies of myopia have been European centric, and the annual sunshine duration varies drastically in Europe, making Europeans ideal subjects for testing our hypothesis.

This study examined the signature of adaptation to myopia-related loci in 1000 Genome Project (1KGP) populations [20], aimed to infer drivers for the differentiation of the genetic basis of myopia. It investigated the role of sunlight exposure in the diversification of myopia prevalence and deepen the understanding of gene-environment interactions in myopia.

## Methods

This study aimed to detect selection signatures in myopia-associated genes within sunlight-related pathways. Given the highly polygenic nature of myopia, we investigated selective sweeps based on allele frequencies and haplotypes, as well as polygenic adaptation based on subtle shifts in allele frequencies within certain pathways. We validated the correlation between the allele frequencies of identified loci and environmental factors and inferred a correlation between sunlight-related exposures and myopia.

### Genotype and summary statistics

The 1KGP phase 3 variant data was downloaded from the International Genome Sample Resource (IGSR) (ftp://ftp.1000genomes.ebi.ac.uk/vol1/ftp/release/20130502/). A set of summary statistics of a meta-genome-wide association study (GWAS) of myopia [21] was downloaded from the FTP site of the King’s College London (ftp://twinr-ftp.kcl.ac.uk/Refractive_Error_MetaAnaly--sis_2020). The GWAS summary statistics used in the two-sample Mendelian randomization [22] were obtained from online sources (https://gwas.mrcieu.ac.uk/) [23].

### Population statistics

Quality control (QC) and formatting of the genotype data were performed using PLINK v.1.9 [24]. The fixation index (*Fst*) was calculated using vcftools [25] (https://vcftools.github.io/man_latest.html). Population branch statistics (PBS) [26] were calculated using pairwise *Fst* in three combinations: Finnish in Finland (FIN), Toscani in Italy (TSI), and Yoruba in Ibadan, Nigeria (YRI); FIN, Bengali in Bangladesh (BEB); and YRI, FIN, Han Chinese in Beijing, China (CHB), and YRI. *nSL* [27] was computed using Selscan 2.0 (https://github.com/szpiech/selscan). Statistical analyses were performed using the R 4.3.0 software, implemented in RStudio 2023.03.1 + 446 (https://www.R-project.org/).

### PBS selection index and detection of polygenic adaptation

A previously described method [28] was applied to detect polygenic adaptation by constructing a SNP-based PBS selection index.

To maximize the inclusion of informative SNPs associated with myopia, we extracted 9927 SNPs from myopia meta-GWAS summary statistics [21] based on linkage disequilibrium (LD) *r*^2^ < 0.1 in 1KGP Europeans (EUR) and *P* < 5.0 × 10^−3^ for myopia association. These SNPs were annotated to 4006 genes using g:profile [29]. An additional 64 loci, expressed in the retinal layers and associated with common refractive errors [11, 21], were added in cases not captured by the g:profile annotation. After removing redundancies, 4033 genes were identified.

The PBS selection index was computed for 4033 genes to measure the likelihood that the mean PBS of randomly selected SNPs would be greater than that of the observed SNPs of the same number. To address the skewed gene size distribution, genes were binned by SNP counts at a window size of 11, which was the mode of all gene sizes. A permutation test with 100,000 iterations was performed to ensure the robustness of the bin PBS selection index. These indices were adjusted using the false discovery rate (FDR). The per-gene PBS selection index was the mean of the corresponding bins, with a PBS selection index < 0.01 considered significant. This metric was not biased by gene sizes (linear regression, *R* ^2^ =  − 2.4 × 10^−4^, *F*-statistic *P* = 0.8586).

A total of 4033 genes were mapped to Gene Ontology Biological Process (GO BP) terms using QuickGO [30]. GO terms with gene counts below the median (*n* = 16) were excluded, resulting in 302 terms. To minimize redundancy, these terms were clustered using the GOSemSim R package [31] and pruned by cutting a Ward hierarchical clustering tree to a height of 0.5. Overlapping terms were removed by prioritizing gene counts, resulting in 260 GO terms. A two-sample proportion test was used to estimate the probability of nonrandom occurrences of per-gene PBS selection index values < 0.01 for each GO term. Outliers indicate subtle shifts in allele frequencies among the GO BP terms, implying polygenic adaptation.

### Gene-environment correlation

Correlations between allele frequency and environmental factors were assessed by Bayenv [32], who described the likelihood that the outliers of selected allele frequencies relative to a standardized set of allele frequencies were due to the selected environmental effect rather than by chance.

Two-sample Mendelian randomization (MR) analyses were conducted using the R script at https://mrcieu.github.io/TwoSampleMR/articles/introduction.html.

## Results

### Signatures of selection in myopia-associated loci

PBS estimates allelic frequency changes between two populations by incorporating an outgroup population to polarize frequency shifts among the three branches based on pairwise *Fst*. A highly positive PBS suggests the presence of potentially positive selection. In total, 6,404,423 SNPs were derived from the summary statistics of a meta-GWAS conducted on European populations [21] after QC. We retained the 99^th^ percentile from the three PBS sets, FIN-TSI-YRI, FIN-BEB-YRI, and FIN-CHB-YRI (Supplementary Figure S2). We filtered their intersection using a threshold of *P* < 5.0 × 10^−3^ for association with myopia [21] meanwhile including more informative SNPs. This process yielded 535 SNPs, subsequently annotated to genes using g:Profiler [29]. We identified 80 genes associated with myopia and showed high PBS values (Supplementary Table S1).

*nSL* is a modified version of the integrated haplotype score (iHS) that employs the count of segregating sites instead of genomic distance for the haplotype length estimation [27]. This modification enhanced its resilience to variations in recombination rates. When *nSL* value is large (|*nSL*|≥ 2), it signifies positive selection for an SNP of interest. In total, 435 autosomal loci of genome-wide significance from a myopia meta-GWAS [21] were subjected to *nSL* filtering. Fourteen loci, including 53 genes, remained at the top SNP at each locus |*nSL*|≥ 2. These were combined with the 80 genes identified by PBS, resulting in 133 myopia-associated genes showing strong signatures in the positive natural sections (Supplementary Table S1). A gene-list enrichment analysis of these genes was performed using KOBAS-i [33], revealing 19 Kyoto Encyclopedia of Genes and Genomes (KEGG) pathways with corrected *P* < 0.05 (Table 1). Notably, the KEGG pathway term “phototransduction” piqued our interest. The input genes for the enrichment analysis influencing phototransduction were derived from *RHO* (rs7984 and rs2855558) and *PDE6G/TSPAN10* (rs9747347), and the expected population differences in allele frequencies were observed, as summarized in Table 2.

**Table 1:** Significant terms of gene enrichment analysis

**KEGG ID**	**KEGG term**	**Enrichment corrected *P***
hsa05416	Viral myocarditis	5.94E-03
hsa04612	Antigen processing and presentation	7.48E-03
hsa05321	Inflammatory bowel disease (IBD)	3.59E-02
hsa04145	Phagosome	3.59E-02
hsa05150	*Staphylococcus aureus* infection	3.59E-02
hsa05140	Leishmaniasis	3.59E-02
hsa05164	Influenza A	3.59E-02
hsa05152	Tuberculosis	3.98E-02
hsa04964	Proximal tubule bicarbonate reclamation	3.98E-02
hsa04658	Th1 and Th2 cell differentiation	4.16E-02
hsa05169	Epstein-Barr virus infection	4.19E-02
hsa04744	Phototransduction	4.19E-02
hsa05168	Herpes simplex virus 1 infection	4.19E-02
hsa05310	Asthma	4.19E-02
hsa04659	Th17 cell differentiation	4.19E-02
hsa05145	Toxoplasmosis	4.55E-02
hsa04714	Thermogenesis	4.60E-02
hsa04152	AMPK signaling pathway	4.75E-02
hsa05330	Allograft rejection	4.75E-02
**GO term**	**GO name**	**Two-sample proportions test *P***
GO:0001889	Liver development	6.75E-09
GO:0007420	Brain development	9.68E-06
GO:0050896	Response to stimulus	3.37E-04
GO:0007608	Sensory perception of smell	5.94E-04
GO:0006357	Regulation of transcription by RNA polymerase II	7.74E-04
GO:0006606	Protein import into nucleus	8.83E-04
GO:0060271	Cilium assembly	1.07E-03
GO:0001933	Negative regulation of protein phosphorylation	1.74E-03
GO:0007368	Determination of left/right symmetry	2.33E-03
GO:0043010	Camera-type eye development	2.33E-03
GO:0001701	In utero embryonic development	3.27E-03
GO:0007601	Visual perception	4.11E-03

**Table 2:** Allele frequencies of highlighted SNPs detected by selective sweeps

Gene	RHO	RHO	TSPAN10
**rsid**	rs7984	rs2855558	rs9747347
**A1/A2**	G/A	G/A	T/C
**A1 effect size**	− 0.01552	− 0.01385	− 0.03339
**GWAS** ***P***-**val**	6.47 × 10^−5^	3.14 × 10^−4^	2.22 × 10^−50^
**FIN nSL**	− 2.58406	− 2.41519	2.0204
**Phenotypic consequence of the selected allele**	Hyperopic	Hyperopic	Myopic
**A1 frequencies in PBS populations**			
** FIN-TSI-YRI**	0.07, 0.23, 0.97	0.07, 0.23, 0.97	0.42, 0.34, 0.07
** FIN-BEB-YRI**	0.07, 0.47, 0.97	0.07, 0.46, 0.97	0.42, 0.20, 0.07
** FIN-CHB-YRI**	0.07, 0.58, 0.97	0.07, 0.58, 0.97	0.42, 0.005, 0.07

A1 represents the effect allele, and A2 represents the alternative allele. The A1 effect size (diopters) and *P*-value were derived from the summary statistics of a myopia genome-wide association study (GWAS) [21]. FIN *nSL* denotes selection intensity, and a positive value indicates the extension of haplotypes favoring A1. Population branch statistics (PBS) were calculated using *FIN* Finnish in Finland, *TSI* Toscani in Italy, *YRI* Yoruba in Ibadan, Nigeria, *BEB* Bengali in Bangladesh; and *CHB* Han Chinese in Beijing, China

**Fig. 1:**
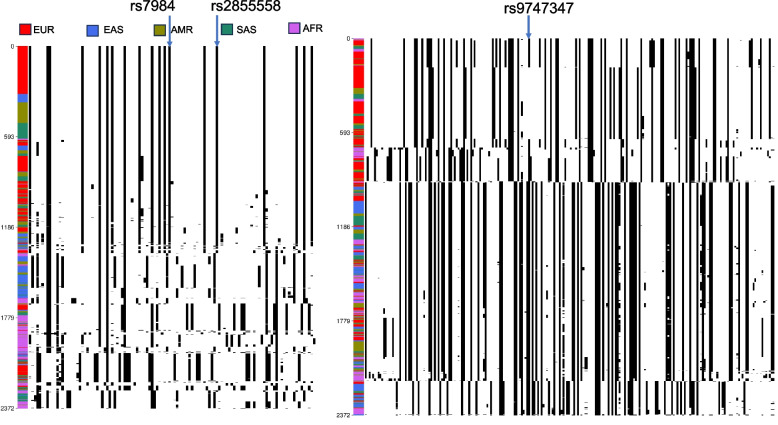
Haplotype distribution of a ~ 30-kb window near the SNPs of interest. Each row represents a haplotype, each column represents an SNP, the black dot indicates a derived allele, and the white dot represents the ancestral allele. The color band on the left indicates the 1KGP population, and the thick group of rows represents a high-frequency haplotype. Left: ~ 30-kb window at *RHO*, including rs7984 and rs2855558. Right: ~ 30-kb window at *TSPAN10*, including rs9747347. EUR, European; EAS, East Asian; AMR, admixed American; SAS, South Asian; AFR, African. The haplotype structures were generated by haplostrips [37]

Rhodopsin, encoded by *RHO*, is responsible for light detection in rod photoreceptor cells. Rhodopsin exhibits significant molecular diversity among mammals based on its ecological niches and behavior [34–36]. The haplotypes of SNPs near rs2855558 and rs7984 (Fig. 1) displayed markedly reduced heterogeneity in the 1KGP Europeans (EUR), mixed Americans (AMR), and South Asians (SAS), whereas East Asians (EAS) and Africans (AFR) exhibited more diverse haplotypes. The cluster of homogeneous haplotypes was predominantly shared by the EUR populations, suggesting strong selection in these populations (Fig. 1).

The haplotypes of *TSPAN10* exhibited a more diverse pattern (Fig. 1), and the EAS and AFR clusters differed from the EUR, AMR, and SAS clusters. Notably, rs9747347 showed a significant association with hair color (*P* = 2.94 × 10 ^−21^) [38]; meanwhile, hair color demonstrates a strong connection to skin pigmentation due to the pleiotropic effect [39]. Given its significant association with hair color and myopia and the selection favoring myopia at this locus (Table 2), we inferred that the selective pressure at *TSPAN10* may originate from adaptation events related to the light environment rather than myopia. Moreover, the two SNPs in *RHO* did not show genome-wide significance in the GWAS summary statistics. Therefore, selective signatures in *RHO* and *TSPAN10* may result from sunlight-related environmental factors, such as pigmentation, rather than selective pressure on myopia (Supplementary Table S2).

### Signatures of polygenic adaptation

Myopia is a complex trait with a highly polygenic architecture [11, 21]. However, methods used for selective sweeps have limited power to detect polygenic adaptations [40, 41]. Using a previously described approach [28], we calculated the PBS selection index for genome-wide myopia-associated genes. Genes with a mean per-gene PBS selection index of less than 0.01 were considered to have constant allele frequency shifts that were not by chance, indicating polygenic selection. These genes were subsequently enriched in GO BP pathways, and we applied a two-sample proportion test to search for polygenic allele frequency shift signatures. We highlighted “camera-type eye development” (GO:0043010, *P* = 2.33 × 10^−3^) and “visual perception” (GO:0007601, *P* = 4.11 × 10^−3^) out of 12 significant GO BP terms (Table 1).

**Fig. 2:**
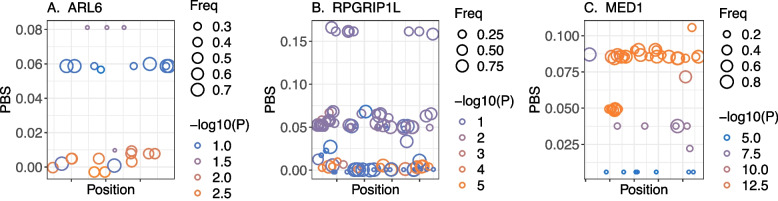
PBS distribution of representative genes in GO term “camera-type eye development” and “visual perception.” **A**, **B**, and **C** illustrate the physical position-dependent distribution of the population branch statistics (PBS) within a 2-kb window upstream and downstream of the *ARL6*, *RPGGRIP1L*, and *MED1* coding region, respectively. PBS values were calculated using the FIN, TSI, and YRI populations. Circles and colors represent allele frequencies and *P*-values of each SNPs, respectively, in a previous myopia GWAS in Europeans [21]. FIN, Finnish in Finland; TSI, Toscani in Italy; YRI, Yoruba in Ibadan, Nigeria

To locate the most significant genes from the identified GO terms, we filtered the genes with a PBS selection index < 0.01, and minimized myopia association *P*-values yielded three loci: *MED1*, *ARL6*, and *RPGRIP1L*. The most significant SNPs in *ARL6* and *RPGRIP1L* were enriched in PBS to close to zero (Fig. 2), indicating that allele frequency shifts in these two genes are less likely to be associated with myopia. Steady allele frequency shifts were enriched in genome-wide significant SNPs in *MED1* (Fig. 2), which serves as a nuclear receptor coactivator and interacts significantly with RNA polymerase II, influencing the expression of protein-coding genes [42]. *MED1* is associated with mammalian circadian rhythms [43–45].

### Gene-environment interactions

Consistent with rs1426654 (*SLC24A5*), a marker for skin pigmentation [18], a robust correlation was found between latitude and allele frequencies of *RHO* across 1KGP populations (Bayes factor = 37.62 ± 2.7; Supplementary Table S2), supporting its interactions with sunlight exposure. The diminished correlation in *TSPAN10* (Bayes factor = 5.72 ± 0.4; Supplementary Table S2) did not negate its interaction with sunlight exposure regarding function and pleiotropic effect from skin pigmentation genes. The inflated Bayes factor for temperature and longitude could result from covariance with latitude. At the same time, the poor relationship with sunshine duration may be due to confounding factors such as measurement methods.

Our two-sample MR analysis (Supplementary Table S3) investigated the relationship between sunlight-related exposure and myopia, as sunlight exposure inhibits the progression of myopia, and adaptive signatures were identified in light-related pathways. The examined exposures included ease of skin tanning, time spent outdoors, and sleep duration. A significant correlation was found between sleep duration and myopia (MR Egger, *P* = 0.01, Supplementary Table S3), with the causal inference that longer sleep leads to a higher risk of myopia. This finding supports the selective signature of *MED1*. However, a significant pleiotropic effect (*P* = 0.0184, Supplementary Table S3) between genes of sleep duration and myopia suggested that this causal relationship could be a false-positive result. Marginally, significant pleiotropy (*P* = 0.0506, Supplementary Table S3) was observed in the time spent outdoors in summer, implying a shared genetic background between myopia and these sunlight-related traits.

## Discussion

School myopia, or simple myopia, is the main form in the recent epidemic. It is typically characterized by a mean spherical equivalent ranging from − 0.5 to − 6 diopters. High myopia, defined as less than − 6 diopters, involves extreme axial elongations and is distinct from school myopia in terms of etiology and genetics [4, 21, 46]. Given that school myopia is the major form of myopia and is largely influenced by environmental factors, our study concentrates on gene-environment interactions, and it should be noted that our findings and implications are specifically applicable to school myopia.

We identified significant selection signatures in myopia-associated genes related to sunlight, including *RHO*, *TSPAN10*, and *MED1*, and we validated the interaction between these genes and the light environment. These findings may provide insights into the genetic basis and diversity of the protective effect of bright sunlight exposure on the risk of myopia incidence.

We proposed *TSPAN10* as evidence of evolutionary mismatch in myopia. We examined the top 64 myopia-associated loci that were expressed in the retinal layers and associated with common refractive errors [11, 21], SNPs representing *TSPAN10*, *KCNJ5*, *TFAP2B*, and *FBN2* had |*nSL*|> 2, which were under strong selection. In contrast, selection favoring the risk allele associated with myopia predisposition was only observed in *TSPAN10* (Table 2) and *FBN2* (*P* = 8.63 × 10^−11^ for rs6860901) [4, 21]. Such loci are considered as evidence of evolutionary mismatches. However, a comprehensive explanation or quantitative approach is lacking to address how these mismatches contribute to the disparity in myopia prevalence.

Most of the top KEGG pathways enriched in myopia-associated genes, with evidence for selective sweeps, were related to communicable diseases and the immune system (Table 1). These identified pathways may be involved in the rapid evolution of immune system during the dispersal of northern Europeans [47]. Myopia is driven by genes participating in the development of all components of the eye [21]. The eye is unique in that it possesses immune privilege [48, 49]; meanwhile, the ocular layer is exposed to the external environment that has developed specific strategies to defend against microbial pathogens [50]. Myopia has also been reported to be related to the immune system, inflammation plays a crucial role in the development of myopia, and HLA genes were reported involving genetic diversity of myopia [51, 52]. Natural selection acting on the immune system, either in the eye or in other organs, may have contributed to the formation of genetic susceptibility to myopia. On the other hand, the outliers of the GO terms in the test for polygenic adaptation (Table 1) are consistent with previous findings that myopia-associated genes are enriched in signal transduction pathways[4, 21]. Meanwhile, PBS was calculated using FIN-TSI-YRI, which reflects traces of recent adaptations of Europeans involving diet, pigmentation, immunity, and body morphology (Table 1), as reported in a previous study [47]. The difference between the two pathway sets is due to the methods used, PBS and *nSL* were used to search for selective sweeps in KEGG pathways, while the PBS selection index was used to search for subtle allele frequency shifts in GO terms. Our results intuitively show that communicable diseases and pathogens are often fatal, while selective pressure from lifestyle changes is mild and long-lasting.

The prevalence of myopia has surged over the past century, paralleling the advent of modern lifestyles. Consequently, this phenotype is unlikely to have been adaptive in ancient environments. There is consensus in the literature that biological pathways related to perception play a critical role in the development of myopia. Signatures of natural selection found at *RHO* and other light perception genes indicate that the evolution of such pathways contributed to shaping the genetic background of myopia. Additionally, the selection signature at *MED1* and the potential causal relationship between sleep duration and myopia may suggest that adaptative changes in the circadian rhythm pathways also participated into this process. The evolution of the genes in these pathways would be related with adaptation to sunlight environment of northern Europe. Moreover, the broad pathways identified in the top selective signatures provide additional evidence that adaptation of the immune system during the migration to Europe has profoundly and systematically impacted the genetic background of myopia. However, it remains unclear which phenotypes natural selection at myopia-related genes favored in ancestral European populations, and selection patterns may have varied depending on local environments over time.

Our inferences were derived exclusively from GWAS summary statistics based on European populations. Comprehensive inference of gene-environment interactions remains challenging owing to insufficient population diversity in Europeans. While we focused mainly on light-induced pathways, morphological and immunological pathways were more robust among outliers, implying an underestimated systematic pleiotropic effect on the genetic basis of myopia.

## Conclusion

We reported significant selection signatures enriched in pathways related to vision and light perception in 1KGP Europeans. These findings suggest that variations in myopia prevalence among populations can be attributed to local adaptation to the light environment and the relevant pleiotropic effects of other biological pathways, such as immune function. We infer that the geographic diversity of the genetic predisposition to myopia is substantial. However, this hypothesis necessitates further corroboration from parallel studies. Notably, GWAS of myopia with power equivalent to those conducted in Europeans are conspicuously lacking in other populations, especially in high susceptibility regions such as East Asia. Our findings contribute to a better understanding of the global disparity in myopia prevalence and provide insights into the implementation of population-targeted strategies from the perspective of evolutionary medicine.

### Supplementary Information

**Additional file 1: Figure S1. **The myopia prevalence distribution. Global myopia prevalence by region. Myopia prevalence records were extracted from previous studies1,2, and re-arranged by continents, comprising 175 studies and 64 countries. **Figure S2.** Population branch statistics (PBS) of myopia-associated SNPs from a myopia GWAS3. (A) PBS of FIN, TSI, and YRI. (B) PBS of FIN, BEB, and YRI. (C) PBS of FIN, CHB, and YRI. FIN. Genotype data from 1000 genome project (1KGP)4. FIN: Finnish in Finland; TSI: Toscani in Italia; BEB: Bengali from Bangladesh; CHB: Han Chinese in Beijing, China; YRI: Yoruba in Ibadan, Nigeria. **Figure S3.** Plots of Two-sample Mendelian Randomization analysis5 between sleep duration and myopia. Analysis results see Table S2. (A) Scatter plot representing effects of exposure (sleep duration) to outcome (myopia). (B) Forest plot, both MR Egger and IVW showing positive correlation of sleep duration with myopia (binary, OR) that longer sleep duration leads to higher risk of myopia. (C) Leave-one-out sensitivity analysis, measures whether the thorough effect was biased by a single SNP of large effect; not all the error bars are larger than 0 stands for influence from SNPs of large effect sizes. **Table S1. **133 gene symbols resulted from KOBAS-i gene-list enrichment analysis. **Table S2.** Correlations of allele frequencies and environmental factors in all 26 1000 Genomes Project populations4. **Table S3.** Two-sample Mendelian randomization analyses in myopia and related factors.

## Data Availability

Not applicable.

## Abbreviations

1KGP: 1000 Genomes Project
GWAS: Genome-wide association study
MR: Mendelian randomization
Fst: Fixation index
PBS: Population branch statistics
FIN: Finnish in Finland
TSI: Toscani in Italy
YRI: Yoruba in Ibadan, Nigeria
BEB: Bengali in Bangladesh
CHB: Han Chinese in Beijing, China
nSL: Number of segregating sites by length
SNP: Single-nucleotide polymorphism
LD: Linkage disequilibrium
EUR: 1KGP Europeans
FDR: False discovery rate
GO BP: Gene Ontology Biological Process
KEGG: Kyoto Encyclopedia of Genes and Genomes
RHO: Rhodopsin
TSPAN10: Tetraspanin 10
EAS: 1KGP East Asian
AMR: 1KGP admixed American
SAS: 1KGP South Asian
AFR: 1KGP African
MED1: Mediator complex subunit 1
ARL6: ADP ribosylation factor like GTPase 6
RPGRIP1L: Retinitis pigmentosa GTPase regulator interacting protein 1
SLC24A5: Solute carrier family 24 member 5
KCNJ5: Potassium inwardly rectifying channel subfamily J member 5
TFAP2B: Transcription factor AP-2 beta
FBN2: Fibrillin 2
HLA: Human leukocyte antigen

## References

[1] Holden BA, Fricke TR, Wilson DA, Jong M, Naidoo KS, Sankaridurg P (2016). Global prevalence of myopia and high myopia and temporal trends from 2000 through 2050. Ophthalmology.

[2] Morgan IG, French AN, Ashby RS, Guo X, Ding X, He M (2018). The epidemics of myopia: aetiology and prevention. Prog Retin Eye Res.

[3] Williams KM, Bertelsen G, Cumberland P, Wolfram C, Verhoeven VJM, Anastasopoulos E (2015). Increasing prevalence of myopia in Europe and the impact of education. Ophthalmology.

[4] Tedja MS, Haarman AEG, Meester-Smoor MA, Kaprio J, Mackey DA, Guggenheim JA (2019). IMI – Myopia genetics report. Invest Ophthalmol Vis Sci.

[5] Cai X-B, Shen S-R, Chen D-F, Zhang Q, Jin Z-B (2019). An overview of myopia genetics. Exp Eye Res.

[6] Xiong S, Sankaridurg P, Naduvilath T, Zang J, Zou H, Zhu J (2017). Time spent in outdoor activities in relation to myopia prevention and control: a meta-analysis and systematic review. Acta Ophthalmol.

[7] Sherwin JC, Reacher MH, Keogh RH, Khawaja AP, Mackey DA, Foster PJ (2012). The association between time spent outdoors and myopia in children and adolescents: a systematic review and meta-analysis. Ophthalmology.

[8] Smith EL, Hung L-F, Huang J (2012). Protective effects of high ambient lighting on the development of form-deprivation myopia in rhesus monkeys. Invest Ophthalmol Vis Sci.

[9] Ashby R, Ohlendorf A, Schaeffel F (2009). The effect of ambient illuminance on the development of deprivation myopia in chicks. Invest Ophthalmol Vis Sci.

[10] Muralidharan AR, Lança C, Biswas S, Barathi VA, Wan Yu Shermaine L, Seang-Mei S, et al. Light and myopia: from epidemiological studies to neurobiological mechanisms. Ophthalmol Eye Dis. 2021;13:25158414211059246. 10.1177/25158414211059246 PMC8721425 34988370

[11] Tedja MS, Wojciechowski R, Hysi PG, Eriksson N, Furlotte NA, Verhoeven VJM (2018). Genome-wide association meta-analysis highlights light-induced signaling as a driver for refractive error. Nat Genet.

[12] Hagen LA, Gjelle JVB, Arnegard S, Pedersen HR, Gilson SJ, Baraas RC (2018). Prevalence and possible factors of myopia in Norwegian adolescents. Sci Rep.

[13] Pärssinen O (2012). The increased prevalence of myopia in Finland. Acta Ophthalmol.

[14] Pärssinen O, Kauppinen M (2022). Associations of near work time, watching TV, outdoors time, and parents’ myopia with myopia among school children based on 38-year-old historical data. Acta Ophthalmol.

[15] Morgan IG, Ohno-Matsui K, Saw S-M (2012). Myopia. The Lancet.

[16] Long E (2018). Evolutionary medicine: why does prevalence of myopia significantly increase?. Evolution, Medicine, and Public Health.

[17] Lea AJ, Clark AG, Dahl AW, Devinsky O, Garcia AR, Golden CD, et al. Evolutionary mismatch and the role of GxE interactions in human disease. arXiv. 2023. http://arxiv.org/abs/2301.05255. Accessed 26 May 2023.

[18] BasuMallick C, Iliescu FM, Möls M, Hill S, Tamang R, Chaubey G (2013). The light skin allele of SLC24A5 in South Asians and Europeans shares identity by descent. PLoS Genet.

[19] Rees JS, Castellano S, Andrés AM (2020). The genomics of human local adaptation. Trends Genet.

[20] Auton A, Abecasis GR, Altshuler DM, Durbin RM, Abecasis GR, Bentley DR (2015). A global reference for human genetic variation. Nature.

[21] Hysi PG, Choquet H, Khawaja AP, Wojciechowski R, Tedja MS, Yin J (2020). Meta-analysis of 542,934 subjects of European ancestry identifies new genes and mechanisms predisposing to refractive error and myopia. Nat Genet.

[22] Hemani G, Zheng J, Elsworth B, Wade KH, Haberland V, Baird D, et al. The MR-base platform supports systematic causal inference across the human phenome. Loos R, editor. eLife. 2018;7:e34408. 10.7554/eLife.34408 PMC5976434 29846171

[23] Elsworth B, Lyon M, Alexander T, Liu Y, Matthews P, Hallett J, et al. The MRC IEU OpenGWAS data infrastructure. bioRxiv. 2020. https://www.biorxiv.org/content/10.1101/2020.08.10.244293v1. Accessed 27 July 2023.

[24] Chang CC, Chow CC, Tellier LC, Vattikuti S, Purcell SM, Lee JJ. Second-generation PLINK: rising to the challenge of larger and richer datasets. GigaScience. 2015;4:s13742–015–0047–8. 10.1186/s13742-015-0047-8 PMC4342193 25722852

[25] Danecek P, Auton A, Abecasis G, Albers CA, Banks E, DePristo MA (2011). The variant call format and VCFtools. Bioinformatics.

[26] Yi X, Liang Y, Huerta-Sanchez E, Jin X, Cuo ZXP, Pool JE (2010). Sequencing of 50 human exomes reveals adaptation to high altitude. Science.

[27] Ferrer-Admetlla A, Liang M, Korneliussen T, Nielsen R (2014). On detecting incomplete soft or hard selective sweeps using haplotype structure. Mol Biol Evol.

[28] Bergey CM, Lopez M, Harrison GF, Patin E, Cohen JA, Quintana-Murci L (2018). Polygenic adaptation and convergent evolution on growth and cardiac genetic pathways in African and Asian rainforest hunter-gatherers. Proc Natl Acad Sci.

[29] Raudvere U, Kolberg L, Kuzmin I, Arak T, Adler P, Peterson H (2019). g:Profiler: a web server for functional enrichment analysis and conversions of gene lists (2019 update). Nucleic Acids Res.

[30] Binns D, Dimmer E, Huntley R, Barrell D, O’Donovan C, Apweiler R (2009). QuickGO: a web-based tool for Gene Ontology searching. Bioinformatics.

[31] Yu G, Li F, Qin Y, Bo X, Wu Y, Wang S (2010). GOSemSim: an R package for measuring semantic similarity among GO terms and gene products. Bioinformatics.

[32] Günther T, Coop G (2013). Robust identification of local adaptation from allele frequencies. Genetics.

[33] Bu D, Luo H, Huo P, Wang Z, Zhang S, He Z (2021). KOBAS-i: intelligent prioritization and exploratory visualization of biological functions for gene enrichment analysis. Nucleic Acids Res.

[34] Gai Y, Tian R, Liu F, Mu Y, Shan L, Irwin DM, et al. Diversified mammalian visual adaptations to bright- or dim-light environments. Mol Biol Evol. 2023;40:msad063. 10.1093/molbev/msad063 PMC10075062 36929909

[35] Dungan SZ, Chang BSW (2022). Ancient whale rhodopsin reconstructs dim-light vision over a major evolutionary transition: implications for ancestral diving behavior. Proc Natl Acad Sci.

[36] Yamaguchi K, Koyanagi M, Sato K, Terakita A, Kuraku S (2023). Whale shark rhodopsin adapted to deep-sea lifestyle by a substitution associated with human disease. Proc Natl Acad Sci.

[37] Marnetto D, Huerta-Sánchez E (2017). Haplostrips: revealing population structure through haplotype visualization. Methods Ecol Evol.

[38] Morgan MD, Pairo-Castineira E, Rawlik K, Canela-Xandri O, Rees J, Sims D (2018). Genome-wide study of hair colour in UK Biobank explains most of the SNP heritability. Nat Commun.

[39] Stern AJ, Speidel L, Zaitlen NA, Nielsen R (2021). Disentangling selection on genetically correlated polygenic traits via whole-genome genealogies. Am J Human Genetics.

[40] Pritchard JK, Di Rienzo A (2010). Adaptation – not by sweeps alone. Nat Rev Genet.

[41] Wellenreuther M, Hansson B (2016). Detecting polygenic evolution: problems, pitfalls, and promises. Trends Genet.

[42] Taatjes DJ (2010). The human mediator complex: a versatile, genome-wide regulator of transcription. Trends Biochem Sci.

[43] Takahashi JS (2017). Transcriptional architecture of the mammalian circadian clock. Nat Rev Genet.

[44] Lande-Diner L, Boyault C, Kim JY, Weitz CJ (2013). A positive feedback loop links circadian clock factor CLOCK-BMAL1 to the basic transcriptional machinery. Proc Natl Acad Sci.

[45] Misra N, Damara M, Ye T, Chambon P (2023). The circadian demethylation of a unique intronic deoxymethylCpG-rich island boosts the transcription of its cognate circadian clock output gene. Proc Natl Acad Sci.

[46] Morgan IG, Rose KA (2019). Myopia: is the nature-nurture debate finally over?. Clin Exp Optom.

[47] Mathieson I, Lazaridis I, Rohland N, Mallick S, Patterson N, Roodenberg SA, et al. Eight thousand years of natural selection in Europe. bioRxiv. 2015. https://www.biorxiv.org/content/10.1101/016477v2. Accessed 27 July 2023.

[48] Jiang LQ, Streilein JW, McKinney C (1994). Immune privilege in the eye: an evolutionary adaptation. Dev Comp Immunol.

[49] Niederkorn JY (2019). The eye sees eye to eye with the immune system: the 2019 Proctor Lecture. Invest Ophthalmol Vis Sci.

[50] Akpek EK, Gottsch JD (2003). Immune defense at the ocular surface. Eye.

[51] Baker RS, Rand LI, Krolewski AS, Maki T, Warram JH, Aiello LM (1986). Influence of HLA-DR phenotype and myopia on the risk of nonproliferative and proliferative diabetic retinopathy. Am J Ophthalmol.

[52] Lin H-J, Wei C-C, Chang C-Y, Chen T-H, Hsu Y-A, Hsieh Y-C, et al. Role of chronic inflammation in myopia progression: clinical evidence and experimental validation. eBioMedicine. 2016;10:269–81. 10.1016/j.ebiom.2016.07.021 PMC5006729 27470424

