# Supplementary material for: Signatures of adaptation in myopia-related genes on the sunlight exposure hypothesis
Source: J Physiol Anthropol. 2023 Nov 2;42:25. doi: 10.1186/s40101-023-00341-4 (PMC10621121; doi:10.1186/s40101-023-00341-4)
Supplement: Supplementary file 1 — Additional file 1: Figure S1. The myopia prevalence distribution. Global myopia prevalence by region. Myopia prevalence records were extracted from previous studies1,2, and re-arranged by continents, comprising 175 studies and 64 countries. Figure S2. Population branch statistics (PBS) of myopia-associated SNPs from a myopia GWAS3. (A) PBS of FIN, TSI, and YRI. (B) PBS of FIN, BEB, and YRI. (C) PBS of FIN, CHB, and YRI. FIN. Genotype data from 1000 genome project (1KGP)4. FIN: Finnish in Finland; TSI: Toscani in Italia; BEB: Bengali from Bangladesh; CHB: Han Chinese in Beijing, China; YRI: Yoruba in Ibadan, Nigeria. Figure S3. Plots of Two-sample Mendelian Randomization analysis5 between sleep duration and myopia. Analysis results see Table S2. (A) Scatter plot representing effects of exposure (sleep duration) to outcome (myopia). (B) Forest plot, both MR Egger and IVW showing positive correlation of sleep duration with myopia (binary, OR) that longer sleep duration leads to higher risk of myopia. (C) Leave-one-out sensitivity analysis, measures whether the thorough effect was biased by a single SNP of large effect; not all the error bars are larger than 0 stands for influence from SNPs of large effect sizes. Table S1. 133 gene symbols resulted from KOBAS-i gene-list enrichment analysis. Table S2. Correlations of allele frequencies and environmental factors in all 26 1000 Genomes Project populations4. Table S3. Two-sample Mendelian randomization analyses in myopia and related factors. [file 40101_2023_341_MOESM1_ESM.docx]

# Supporting Information

Tian Xia, Kazuhiro Nakayama*

Email: knakayama@edu.k.u-tokyo.ac.jp


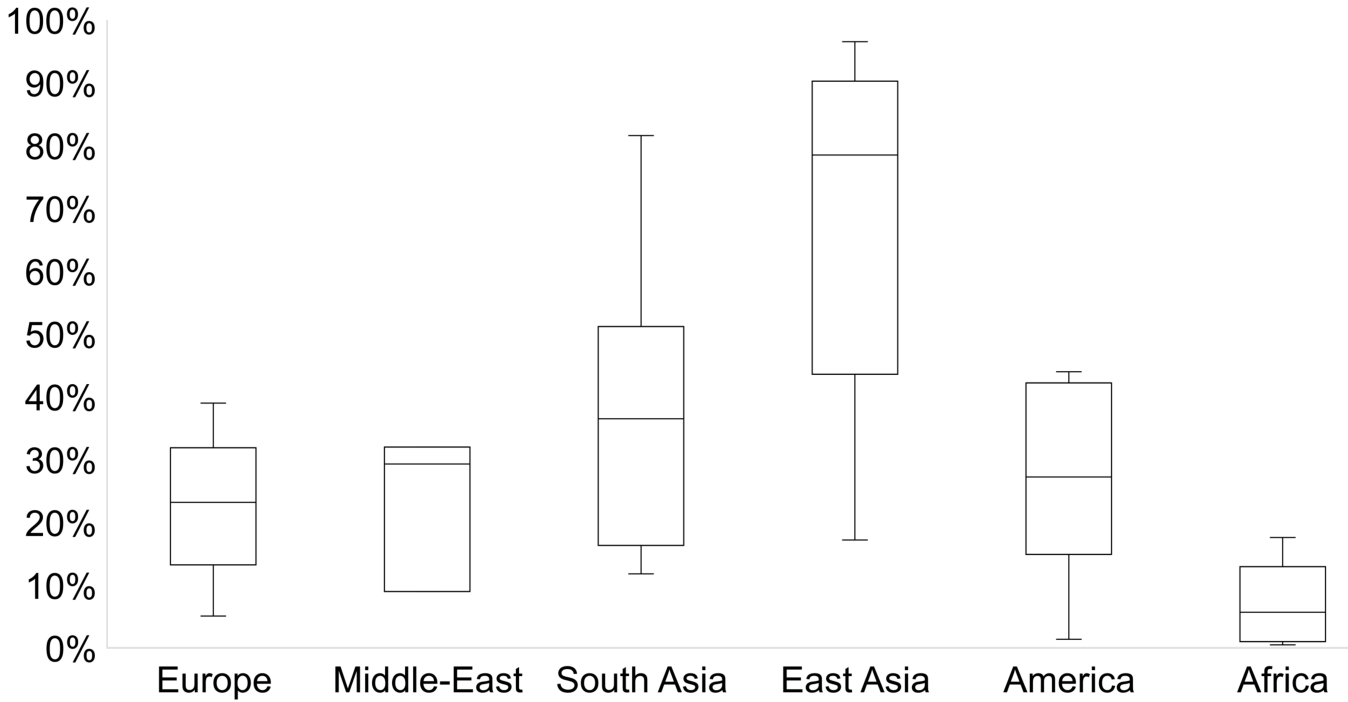


Fig. S1 The myopia prevalence distribution

Global myopia prevalence by region. Myopia prevalence records were extracted from previous studies^1,2^, and re-arranged by continents, comprising 175 studies and 64 countries.

A


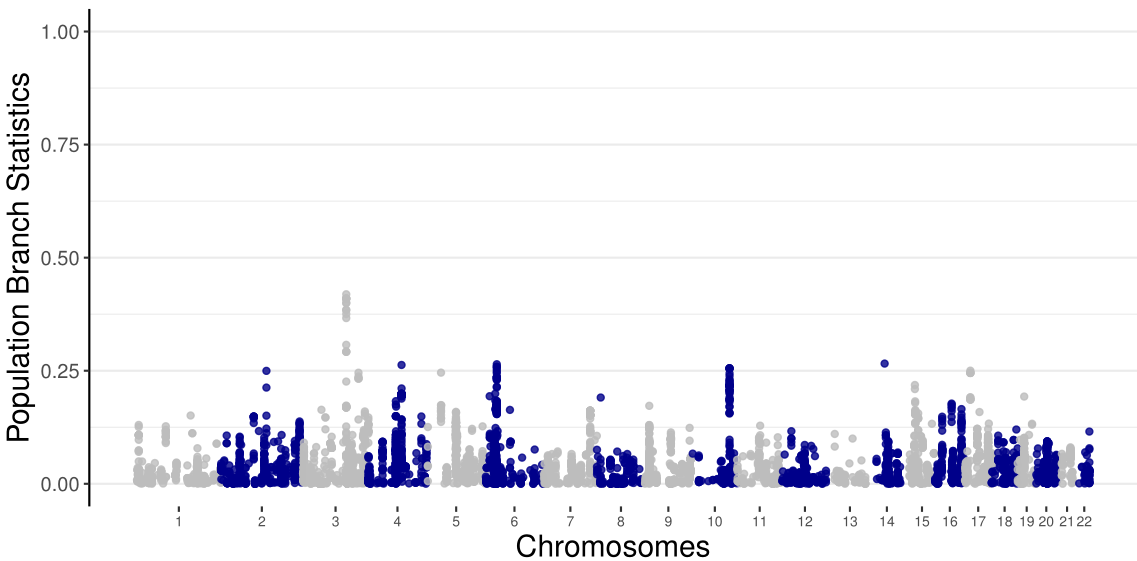


***RHO***

B


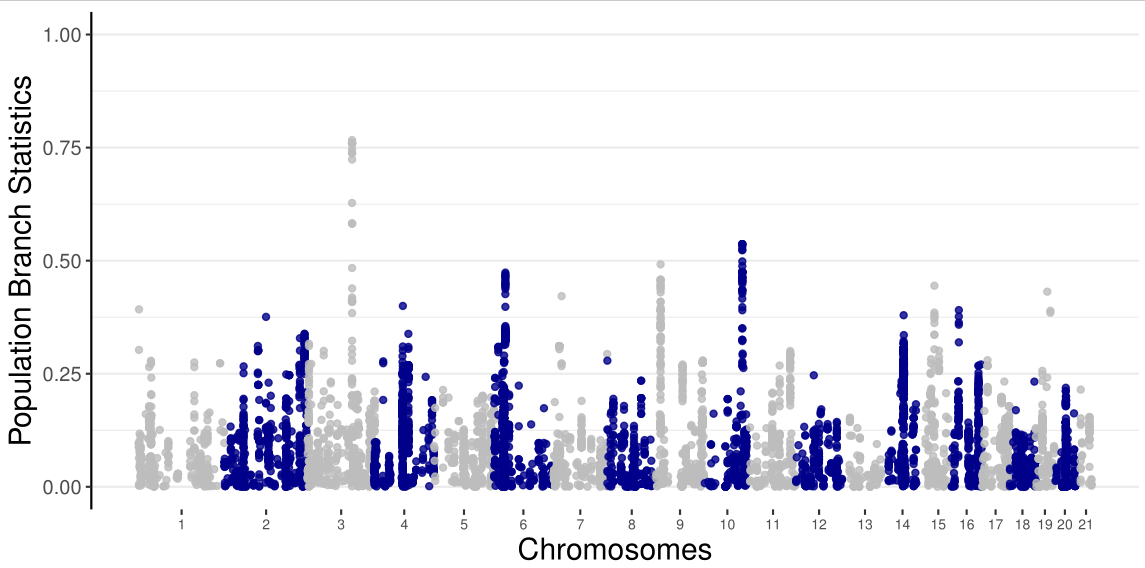


***RHO***

C

***RHO***


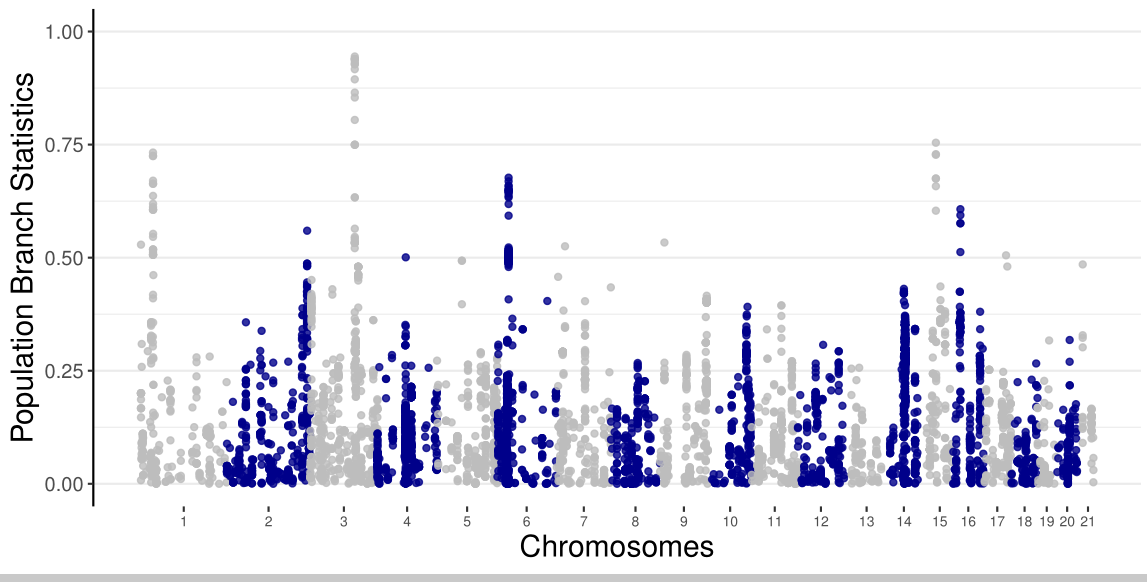


Fig. S2 Population branch statistics (PBS) of myopia-associated SNPs from a myopia GWAS^3^.

(A) PBS of FIN, TSI, and YRI. (B) PBS of FIN, BEB, and YRI. (C) PBS of FIN, CHB, and YRI. FIN. Genotype data from 1000 genome project (1KGP)^4^. FIN: Finnish in Finland; TSI: Toscani in Italia; BEB: Bengali from Bangladesh; CHB: Han Chinese in Beijing, China; YRI: Yoruba in Ibadan, Nigeria.

A.


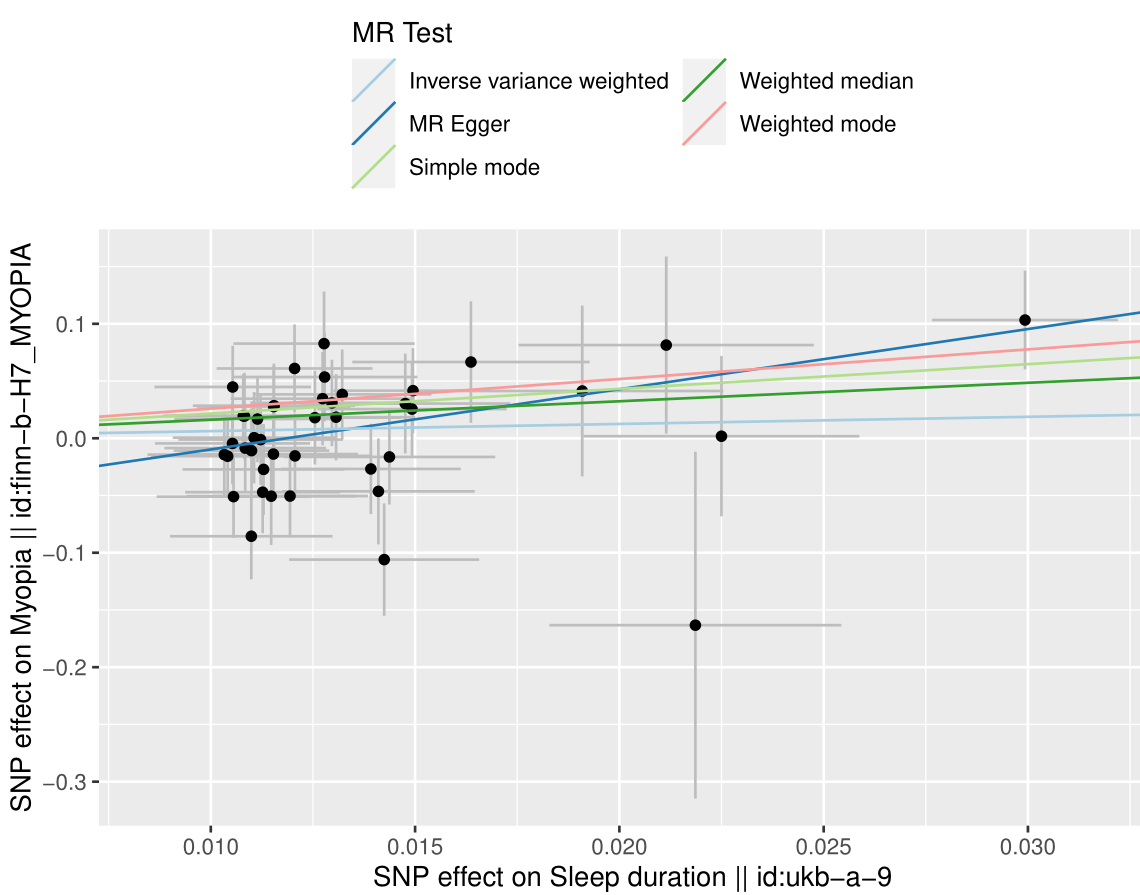


B.


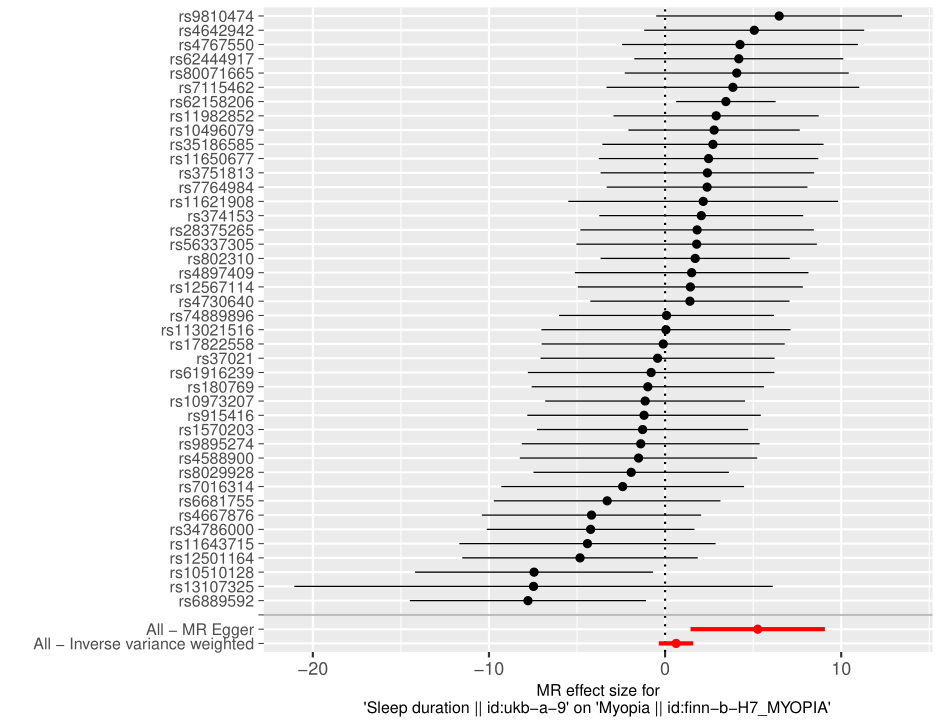


C.


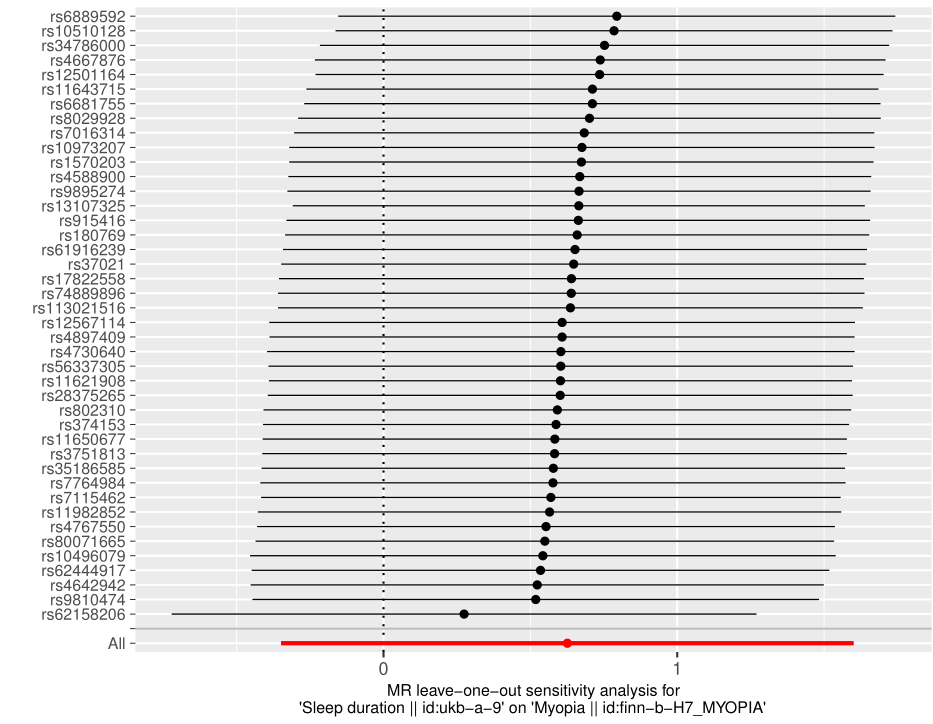


Fig. S3 Plots of Two-sample Mendelian Randomization analysis^5^ between sleep duration and myopia.

Analysis results see Table S2. (A) Scatter plot representing effects of exposure (sleep duration) to outcome (myopia). (B) Forest plot, both MR Egger and IVW showing positive correlation of sleep duration with myopia (binary, OR) that longer sleep duration leads to higher risk of myopia. (C) Leave-one-out sensitivity analysis, measures whether the thorough effect was biased by a single SNP of large effect; not all the error bars are larger than 0 stands for influence from SNPs of large effect sizes.

Table S1. 133 gene symbols resulted from KOBAS-i gene-list enrichment analysis.

| ACOT13 | ENSG00000227033 | L3MBTL4 | RHO |
| --- | --- | --- | --- |
| ACTG1 | ENSG00000229262 | LHX4 | RHPN2 |
| ACTR1B | ENSG00000244932 | LINC00862 | SETBP1 |
| ADAL | ENSG00000253170 | LINC01876 | SLC14A2 |
| ADD3 | ENSG00000254733 | LPAR2 | SLC16A3 |
| ADD3-AS1 | ENSG00000258670 | LRRC45 | SLC22A23 |
| AKAP6 | ENSG00000266846 | LURAP1L-AS1 | SLC25A10 |
| ANKRD36B | ENSG00000267059 | MASP2 | SSU72 |
| ARL16 | ENSG00000267131 | MAU2 | STAT1 |
| ASAH2 | ENSG00000276302 | MBD3 | SUGP1 |
| ATP13A1 | ENSG00000277315 | ME3 | SUMF1 |
| BACH2 | ENSG00000285253 | MEX3D | SYT14 |
| BAHCC1 | ENSG00000285331 | MGAT5 | TARDBP |
| BCAS3 | ENSG00000286121 | MRPL12 | TCF12 |
| BEGAIN | ENSG00000287720 | MYO5C | TMEM105 |
| BOD1 | ENSG00000290993 | NCAN | TOR1AIP1 |
| C14orf39 | FASN | NDUFA13 | TOR1AIP2 |
| C1GALT1 | FBN2 | NKX2-2 | TP53BP1 |
| C2orf92 | FSCN2 | NPLOC4 | TSPAN10 |
| CADM1 | GATAD2A | OR12D3 | TSSK6 |
| CCDC137 | GLS | OR2J3 | TUBGCP4 |
| CCDC57 | GMIP | OR5V1 | VWA3A |
| CDH9 | GPATCH1 | OXLD1 | WDR25 |
| CILP2 | GPC6 | PAPPA | YJEFN3 |
| COPB2 | H1-8 | PBX4 | ZCCHC8 |
| COPB2-DT | HACD3 | PDE6G | ZIC1 |
| DCXR | HAPLN4 | PDIA3 | ZNF101 |
| DIS3L2 | HGS | PGBD1 | ZNF14 |
| DLK1 | HLA-DPA1 | PPARGC1A | ZSCAN26 |
| DPP8 | HLA-DPB1 | PRKG2 | ZSCAN29 |
| DRD1 | IFT122 | PRKG2-AS1 | ZSCAN31 |
| DUS1L | IGDCC4 | PSMG4 |  |
| EEF2K | INTS14 | RAC3 |  |
| EFCAB12 | KCNQ5 | RFXANK |  |

Table S2. Correlations of allele frequencies and environmental factors in all 26 1000 Genomes Project populations^4^

| Bayes factor | Sunshine duration | \|Latitude\| | Mean temperature | Longitude |
| --- | --- | --- | --- | --- |
| rs7984, *RHO* | 1±0.04 | 37.62±2.7 | 13.17±1.18 | 0.95±0.03 |
| rs2855558, *RHO* | 0.52±0.03 | 7.23±0.5 | 7.24±0.52 | 0.34±0.01 |
| rs9747347, *TSPAN10* | 0.14±0.001 | 5.72±0.4 | 0.24±0.003 | 0.32±0.004 |
| rs1426654, *SLC24A5* | 1.06±0.08 | 104.57±20.51 | 0.24±0.01 | 18.86±4.31 |

Correlations analysis between allele frequency and environmental factors were estimated by bayenv^6^. Correlations were verified by bayes factors as value larger than 10 referring confident evidence. Sunshine duration data (hours/year) and annual mean temperatures were retrieved from the Hong Kong Observatory (https://www.hko.gov.hk).

Table S3. Two-sample Mendelian randomization analyses in myopia and related factors.

| **Ease of skin tanning (ukb-b-533, MRC-IEU)**  **myopia (finn-b-H7_MYOPIA, FinnGene)** | **BETA** | **SE** | **P** |
| --- | --- | --- | --- |
| MR Egger | 0.0521 | 0.1063 | 6.25E-01 |
| Weighted median | 0.1466 | 0.1347 | 2.76E-01 |
| Inverse variance weighted | 0.1153 | 0.0873 | 1.86E-01 |
| Simple mode | 0.0621 | 0.2593 | 8.11E-01 |
| Weighted mode | 0.1028 | 0.0981 | 2.97E-01 |
|  | egger_intercept | SE | P |
| pleiotropy | 0.0056 | 0.0054 | 3.00E-01 |
| **Time spend outdoors in summer (ukb-b-969, MRC-IEU)**  **myopia (finn-b-H7_MYOPIA, FinnGene)** | **BETA** | **SE** | **P** |
| MR Egger | 3.5020 | 2.0560 | 9.63E-02 |
| Weighted median | -0.2153 | 0.6659 | 7.47E-01 |
| Inverse variance weighted | -0.5403 | 0.4637 | 2.44E-01 |
| Simple mode | 0.1456 | 1.5146 | 9.24E-01 |
| Weighted mode | 0.7768 | 1.4025 | 5.83E-01 |
|  | egger_intercept | SE | P |
| pleiotropy | -0.0576 | 0.0286 | 5.06E-02 |
| **Time spend outdoors in winter (ukb-b-6811, MRC-IEU)**  **myopia (finn-b-H7_MYOPIA, FinnGene)** | **BETA** | **SE** | **P** |
| MR Egger | 4.6923 | 16.9044 | 8.07E-01 |
| Weighted median | -0.2025 | 2.3647 | 9.32E-01 |
| Inverse variance weighted | -1.2136 | 1.9489 | 5.33E-01 |
| Simple mode | 0.4760 | 3.4654 | 8.99E-01 |
| Weighted mode | 0.4760 | 2.8087 | 8.76E-01 |
|  | egger_intercept | SE | P |
| pleiotropy | -0.0594 | 0.1686 | 7.58E-01 |
| **Sleep duration (ukb-a-9, Neale Lab)**  **myopia (finn-b-H7_MYOPIA, FinnGene)** | **BETA** | **SE** | **P** |
| MR Egger | 5.2606 | 1.9456 | 1.00E-02 |
| Weighted median | 1.6166 | 0.6857 | 1.84E-02 |
| Inverse variance weighted | 0.6259 | 0.4974 | 2.08E-01 |
| Simple mode | 2.1584 | 1.7378 | 2.21E-01 |
| Weighted mode | 2.5881 | 1.2356 | 4.24E-02 |
|  | egger_intercept | SE | P |
| pleiotropy | -0.0624 | 0.0254 | 1.84E-02 |

Scores were calculated by two-sample MR package^5^. All GWAS data from online server <https://gwas.mrcieu.ac.uk/>, named by “Trait (GWAS ID, Consortium)” and has all outcomes as myopia. Causal inference is confident only when all effect sizes (BETA) are larger or smaller than 0, hence only sleep duration shows causal relation with myopia (*P*<0.05, MR Egger when pleiotropy is <0.05^7^).

SI References

1. Holden BA, Fricke TR, Wilson DA, et al. Global Prevalence of Myopia and High Myopia and Temporal Trends from 2000 through 2050. *Ophthalmology*. 2016;123(5):1036-1042. doi:10.1016/j.ophtha.2016.01.006

2. Morgan IG, Ohno-Matsui K, Saw SM. Myopia. *The Lancet*. 2012;379(9827):1739-1748. doi:10.1016/S0140-6736(12)60272-4

3. Hysi PG, Choquet H, Khawaja AP, et al. Meta-analysis of 542,934 subjects of European ancestry identifies new genes and mechanisms predisposing to refractive error and myopia. *Nat Genet*. 2020;52(4):401-407. doi:10.1038/s41588-020-0599-0

4. Auton A, Abecasis GR, Altshuler DM, et al. A global reference for human genetic variation. *Nature*. 2015;526(7571):68-74. doi:10.1038/nature15393

5. Hemani G, Zheng J, Elsworth B, et al. The MR-Base platform supports systematic causal inference across the human phenome. Loos R, ed. *eLife*. 2018;7:e34408. doi:10.7554/eLife.34408

6. Günther T, Coop G. Robust Identification of Local Adaptation from Allele Frequencies. *Genetics*. 2013;195(1):205-220. doi:10.1534/genetics.113.152462

7. Burgess S, Thompson SG. Interpreting findings from Mendelian randomization using the MR-Egger method. *Eur J Epidemiol*. 2017;32(5):377-389. doi:10.1007/s10654-017-0255-x
